# Beaver tail liver and congenital asplenia in a polytrauma patient

**DOI:** 10.11604/pamj.2024.47.18.42531

**Published:** 2024-01-17

**Authors:** Ahmad Alrahmani, Sharfuddin Chowdhury

**Affiliations:** 1 Trauma and Acute Care Surgery Unit, Department of Surgery, College of Medicine, King Saud University, Riyadh, Saudi Arabia,; 2 Trauma Center, King Saud Medical City, Riyadh, Saudi Arabia

**Keywords:** Beaver, liver, polytrauma

## Image in medicine

A 22-year-old male involved in a motor vehicle accident presented to the Emergency Department of King Saud Medical City with facial, neck, and chest injuries. He did not have any previous medical or surgical history. He was conscious, oriented, and vitally stable upon presentation. According to the trauma survey protocol, he underwent a pan-computed tomography (CT) scan following the initial evaluation. A CT scan of the face revealed a nondisplaced fracture of the left palatine bone, a fracture of the left infratemporal wall of the maxillary sinus, and associated hemosinuses. A CT of the neck revealed a C2 fracture involving both lateral masses and the body. A CT of the abdomen revealed an absent spleen and an elongated left lobe of the liver that extended laterally, known as “Beaver tail liver,” and occupied the space of the spleen in the left upper quadrant of the abdomen. As an anatomical variant, he also possessed a double inferior vena cava (IVC) with retro aortic right renal vein and hemi azygos continuation of the IVC. Except for thrombocytosis with a platelet count of 606 X 10^9^/L (reference range, 150 X 10^9^/L to 400 X 10^9^/L), his initial blood results were within normal limits. His peripheral blood smear revealed Howell-Jolly's bodies and a mild thrombocytosis. His facial injury was treated conservatively, while his cervical injury was immobilized with a halo. On the seventh post-admission day, he was discharged after applying a halo vest and attending an outpatient clinic appointment.

**Figure 1:**
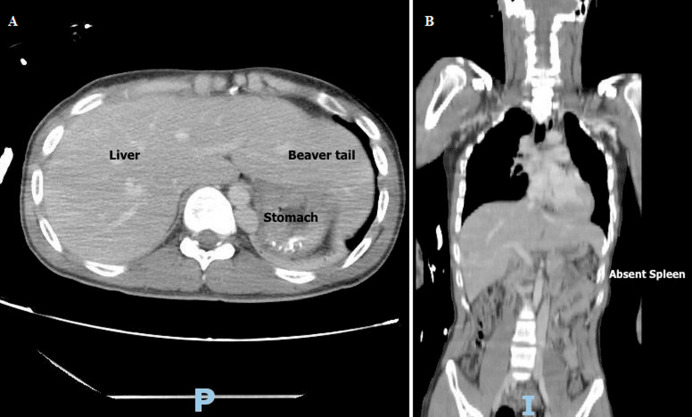
(A,B) axial and coronal views of the CT scan showing a beaver tail liver and asplenia

